# Lifetime encoding in flow cytometry for bead-based sensing of biomolecular interaction

**DOI:** 10.1038/s41598-020-76150-x

**Published:** 2020-11-10

**Authors:** Daniel Kage, Katrin Hoffmann, Heike Borcherding, Uwe Schedler, Ute Resch-Genger

**Affiliations:** 1 Division Biophotonics (BAM-1.2), Federal Institute for Materials Research and Testing (BAM), Richard-Willstätter-Str. 11, 12489 Berlin, Germany; 2 PolyAn GmbH, Rudolf-Baschant-Str. 2, 13086 Berlin, Germany; 3 Department of Physics, Humboldt-Universität zu Berlin, Newtonstr. 15, 12489 Berlin, Germany; 4 Present Address: German Rheumatism Research Centre Berlin (DRFZ) - Flow Cytometry Core Facility, Charitéplatz 1 (Virchowweg 12), 10117 Berlin, Germany

**Keywords:** High-throughput screening, Fluorescence spectroscopy

## Abstract

To demonstrate the potential of time-resolved flow cytometry (FCM) for bioanalysis, clinical diagnostics, and optically encoded bead-based assays, we performed a proof-of-principle study to detect biomolecular interactions utilizing fluorescence lifetime (LT)-encoded micron-sized polymer beads bearing target-specific bioligands and a recently developed prototype lifetime flow cytometer (LT-FCM setup). This instrument is equipped with a single excitation light source and different fluorescence detectors, one operated in the photon-counting mode for time-resolved measurements of fluorescence decays and three detectors for conventional intensity measurements in different spectral windows. First, discrimination of bead-bound biomolecules was demonstrated in the time domain exemplarily for two targets, Streptavidin (SAv) and the tumor marker human chorionic gonadotropin (HCG). In a second step, the determination of biomolecule concentration levels was addressed representatively for the inflammation-related biomarker tumor necrosis factor (TNF-α) utilizing fluorescence intensity measurements in a second channel of the LT-FCM instrument. Our results underline the applicability of LT-FCM in the time domain for measurements of biomolecular interactions in suspension assays. In the future, the combination of spectral and LT encoding and multiplexing and the expansion of the time scale from the lower nanosecond range to the longer nanosecond and the microsecond region is expected to provide many distinguishable codes. This enables an increasing degree of multiplexing which could be attractive for high throughput screening applications.

## Introduction

Flow cytometry (FCM) is a high-throughput screening (HTS) method for the optical analysis of a large number of either single cells or microbead populations in a flow^1,2^. This multiparametric fluorescence technique commonly relies on fluorophores for optical encoding and analyte quantification through spectral and intensity information^3–7^ and is well-established in many analytical and research applications in medical diagnostics, biology, and food analysis^8–11^. FCM is particularly attractive for the readout of multiplexed bioassays and the HTS of biomarkers e.g. in bead-based suspension assays to simultaneously address and quantify various biomarkers^9,12,13^. Conventional FCM instruments are designed to identify biomolecules and to study ligand binding and biomolecular interactions by evaluating signals from fluorescent labels in the intensity domain in different spectral windows^14–18^.

FCM applications linked to bead-based assays require encoding schemes that provide a large number of codes to enable highly parallelized analyses^9,12,13^. Typical encoding schemes utilize spectral and/or intensity codes. Spectral codes can, however, suffer from spectral overlap of the emission bands of encoding luminophores, limiting the number of distinguishable codes and often requiring signal compensation and cross talk corrections^10,19^. Intensity encoding is subject to leaking and photobleaching of the encoding fluorophores and is affected by fluctuations in the excitation light intensity which can lead to systematic errors in code assignment. To increase the number of distinguishable codes in FCM and circumvent these possible drawbacks of spectral multiplexing in the intensity domain, the characteristic fluorescence lifetime (LT) of a fluorophore can be exploited as an additional detection or encoding parameter. An additional advantage of LT measurements is the independence of LT on fluorophore concentration and excitation light intensity which, together with decreasing costs of pulsed light sources and fast detectors, renders time-resolved fluorescence detection increasingly attractive for imaging and sensing applications^4,10,20–22^. LT-FCM has already been considered before as well^5^, and was mostly addressed by combining FCM with fast frequency domain LT techniques compatible with the high-throughput conditions in FCM^5,6,22–27^. LT measurements in the time domain, however, are superior since they are more sensitive and offer a better time resolution as demonstrated for different fluorophores and time scales^22,28^. Nevertheless, this detection technique has been very rarely utilized in FCM as the short interaction time in the flow results in reduced photon count numbers emitted from the objects of interest and hence poorer photon statistics compared to conventional LT measurements^22,29–31^. To tackle this challenge, we recently reported on a prototype LT-FCM designed for intensity and LT measurements in flow using a single excitation light source. In this context, also LT encoding of carrier beads with organic dyes and II/VI semiconductor quantum dots with sufficiently different LTs and LT-based bead classification were shown^31,32^.

Here, we present a proof-of-principle study utilizing LT-encoded fluorophore-loaded micron-sized polymer beads bearing target-specific bioligands to detect and quantify biomolecular interactions with an LT-FCM instrument operated in the time domain. First, discrimination of bead-bound biomolecules is demonstrated for LT-encoded beads surface-functionalized with exemplarily chosen bioligands, here, the biomolecule Streptavidin (SAv) and the tumor marker human chorionic gonadotropin (HCG). Secondly, classification of different concentration levels of biomolecules is shown representatively for the inflammation-related biomarker tumor necrosis factor (TNF-α) by reading out the fluorescence intensity of the so-called ligand fluorescence of a reporter dye in a different spectral window. Assessing the performance of the analytical procedure and providing general quality parameters of bioassays^33^ such as dynamic range of detection, analytical sensitivity and selectivity, specificity, cross-reactivity, reproducibility etc. was beyond this proof-of-concept demonstration.

## Materials and methods

### Materials

Fluorophore-stained, monodisperse poly(methyl methacrylate) (PMMA) microbeads with protein A/G surface modification were provided by PolyAn GmbH. To realize LT encoding with three different LT codes, the dyes PolyAn Red5 (code A) and PolyAn Red (code C) and a combination of dyes Red5 and Red (code B) were sterically incorporated in the beads during particle synthesis, yielding the commercially available LT-encoded polymer beads with diameters of 6.5 µm (code A), 6.3 µm (code B), and 8.8 µm (code C). LT-based discrimination of these three LT codes was previously demonstrated^31^.

For biomolecule sensing and detection, the human anti-chorionic gonadotropin (α + ß) subunit (HCG) capture antibodies in whole serum (Sigma-Aldrich) and HCG (Sigma-Aldrich), HCG detector antibodies labelled with AlexaFluor488 (invitrogen) as well as the streptavidin (SAv) system with SAv capture antibodies (antibodies-online) and SAv labelled with AlexaFluor488 (life technologies) were employed without purification. The fluorescence label AlexaFluor488 was chosen, because its conjugation to e.g. biomolecules results in only minor changes of its spectral properties and the slightly bathochromically shifted emission profiles of the bioconjugates closely resemble that of the free dye^34,35^. For demonstrating the determination of biomolecule concentration levels, beads decorated with anti-human TNF-α polyclonal antibodies P300A (ThermoFisher Scientific), Human TNF-α (protein, Invitrogen), and an anti-human TNF-α monoclonal antibody (Mab11)-Alexa Fluor488 conjugate (ThermoFisher Scientific) were used. For dilution of the bead suspensions and biochemical reagents, three different buffers were employed: buffer 1 (20 mmol/L Na-phosphate buffer, pH8), buffer 2 (buffer 1 + 0.02% (w/v) Tween20 (ThermoFisher Scientific)), and buffer 3 (buffer 1 + 0.001% (w/v) Tween20).

### Sample preparation

For the study on biomolecule interaction sensing with the analytes HCG and SAv seven bead suspensions were made: A1 and C1 (positive control samples), A2 and C2 (negative control samples), A3 and C3 (basis for mixed sample M), and B (additional LT code). First, 500 µL bead suspension (1–2 mg beads, corresponding to ca. 5 × 10^6^ beads, depending on bead size) in buffer 1 were prepared for all samples. Subsequently, 20 µL HCG capture antibody solution (whole serum, 7.5 mg/mL) were added to samples A1, A2, and A3, and 20 µL SAv capture antibody solution (1.35 mg/mL) were added to samples C1, C2, and C3. All samples were incubated for 1 h under agitation (50 rpm, Rotator NeoLab) at room temperature. All samples were washed by centrifugation, removal of the supernatant, and resuspension cycles. This included twice washing with buffer 1 and once with buffer 3 followed by resuspension in buffer 1.

Subsequently, 20 µL HCG antigen solution (0.07 mg/mL) were added to sample A1; and 20 µL buffer 1 were added to samples C1, A2, and C2, respectively. For the mixed sample M, about half of the washed samples A3 and C3 were combined resulting in sample M. Then, 10 µL HCG antigen solution (0.07 mg/mL) and 10 µL buffer 1 were added to sample M. All samples were incubated for 0.5 h under agitation. Subsequently, the samples were washed by centrifugation, removal of the supernatant, and resuspension cycles. This included once washing with buffer 1, twice with buffer 2, once again with buffer 1 followed by resuspension in buffer 1. In the third step, 20 µL HCG detection antibody solution (0.3 mg/mL) were added to samples A1 and A2, and 20 µL SAv solution (0.95 mg/mL) were added to sample C1; 10 µL HCG detector antibody solution (0.3 mg/mL) and 10 µL of a solution of SAv labelled with AlexaFluor488 (0.95 mg/mL) were added to sample M, followed by incubation under agitation (0.5 h). All samples were washed by centrifugation, removal of the supernatant, and resuspension cycles. This included once washing with buffer 3, twice with buffer 1 followed by resuspension in buffer 1.

For the exemplary study on analyte quantification using TNF-α, six bead samples were employed: A0, B0, and C0 as negative controls and AM, BL, CH for medium (AM), low (BL), and high (CH) TNF-α concentration. The preparation procedure was again divided into three parts. First, 1 mg (5 × 10^6^) beads were suspended in 660 µl of buffer 1, and 6.5 µl of TNF-α polyclonal antibody (1 mg/mL) were added to each bead suspension (A0, B0, C0, AM, BL, CH). These samples were incubated under agitation (1 h at room temperature). All samples were washed by centrifugation, removal of the supernatant, and resuspension cycles. This included twice washing with buffer 1, once with buffer 3 followed by resuspension in buffer 1. In the second processing step, 0.5 µl (sample BL), 5 µl (sample AM), or 50 µl (sample CH) solution containing TNF-α (0.01 mg/mL) were added, and the sample volumes were adapted with buffer 1. Buffer 1 was added to samples A0, B0, C0. The samples were incubated under agitation (0.5 h at room temperature). Subsequently, all samples were washed by centrifugation, removal of the supernatant, and resuspension cycles. This included once washing with buffer 1, twice with buffer 2, once again with buffer 1 followed by resuspension in buffer 1. In the third step, 5 µl Mab11-AlexaFluor 488 (0.1 mg/mL) as detection antibody for TNF-α were added to all samples and the samples were incubated under agitation (1 h). All samples were washed by centrifugation, removal of the supernatant, and resuspension cycles. This included once washing with buffer 2, twice with buffer 1 followed by resuspension in buffer 1.

### Steady-state fluorescence measurements on ensembles of beads in suspension

The fluorescence spectra of ensembles of beads in suspension were measured with the calibrated spectrofluorometers FSP920 and FLS920 (Edinburgh Instruments Ltd.)^31,32^. We used an L-geometry setup of the excitation and emission channel and magic angle^1^ polarizer settings. The emission spectra were corrected for the wavelength-dependent spectral responsivity of the detection channel. The spectral bandpass for measuring the emission spectra was set to 4–6 nm. Random fluctuations of the light source intensity were considered with a reference detector.

### Confocal laser scanning microscopy (CLSM)

Single-particle microscopy measurements were performed with beads suspended in water (milliQ) and transferred onto a coverslip. The microscopy images were recorded with a FluoView FV1000 microscope (Olympus GmbH, Germany), similar to the procedure described earlier^31,32,36^. A multiline argon ion laser (488 nm, 30 mW) and a green HeNe laser (543 nm, 1 mW) were used as excitation light sources. The excitation light was reflected by a dichroic mirror DM 488/543/633 and focused onto the sample through an Olympus objective UPLSAPO 60 × W (numerical aperture N.A. 1.2). The emitted photons were collected with the same objective. The different spectral channels were defined by optical filters (detection settings: channel 1 was defined by an emission dichroic mirror SDM560 combined with a variable band pass filter position 495 nm and a filter range of 100 nm, and channel 2 by a barrier filter BA650IF).

### Time-resolved fluorescence measurements on ensembles of beads in suspension

The standard time-resolved measurements were performed with a lifetime spectrometer FLS920 (Edinburgh Instruments Ltd.) equipped with a Fianium Supercontinuum SC400-2-PP (NKT Photonics A/S) and a Hamamatsu R3809U-50 (Hamamatsu Photonics K.K.) MCP-PMT in standard time-correlated single-photon counting mode^31,32^. The instrument response function, obtained with a Ludox suspension, has a pulse width of about 250 ps. The repetition rate for excitation was set to 10 MHz. Magic angle polarizer settings were applied. The measured fluorescence decay kinetics were evaluated using the reconvolution procedure of the FAST program (Edinburgh Instruments Ltd.). From the measured, multi-exponential decays, the intensity-weighted average lifetimes τ_int_ were determined from least-squares multi-exponential decay fits of the data.

For all ensemble measurements of beads in suspension, standard 1 cm quartz cuvettes (Hellma GmbH & Co. KG) were used. The bead suspensions were continuously stirred during the measurements. All measurements were carried out at room temperature (≈20 °C).

### Lifetime flow cytometry (LT-FCM) setup

FCM measurements with nanosecond time resolution were performed with a Quantum P/pantau cytometer (Quantum Analysis GmbH) recently developed^31^. The setup is equipped with 488 nm laser diode for excitation that was intensity-modulated with a 5-MHz square wave. Three channels operated in conventional low-bandwidth mode were used for the detection of scattered light (side scatter, SSC) and fluorescence photons (FL1, ‘green’ channel for ligand fluorescence with 520(14) nm bandpass filter; FL2 with a 530 nm long-pass filter). These channels only provide fluorescence intensity values. Additionally, a ‘red’ LT detection channel for time-resolved measurements of fluorescence decays was operated in the photon counting mode and defined by a 620 nm dichroic mirror. The samples were diluted in order to reach concentrations of 10^4^–10^5^ beads per mL.

### Data analysis

Time-resolved data from flow cytometry were used to determine luminescence LTs on the single-particle level. The luminescence decay curves are histograms of photon arrival times with a bin width of 2.5 ns. From each luminescence intensity decay curve, a LT value was determined by means of Eq. (1)^1^.

1 $$\tau_{{{\text{mean}}}} = \frac{{\mathop \smallint \nolimits_{0}^{\theta } tI\left( t \right){\text{d}}t}}{{\mathop \smallint \nolimits_{0}^{\theta } I\left( t \right){\text{d}}t}} \approx \frac{{\mathop \sum \nolimits_{j = 1}^{{j_{\max } }} t_{j} I_{j} }}{{\mathop \sum \nolimits_{j = 1}^{{j_{max} }} I_{j} }}$$

Here $I\left( t \right)$ is the luminescence intensity at time $t$ and $\theta$ limits the time range considered for lifetime determination. Due to the discrete nature of the data, the integral is replaced by a sum over the respective bins such that $t_{j}$ denotes the time value associated with a bin and $I_{j}$ is the respective number of collected fluorescence photons in that bin. Data analysis was carried out with custom-made scripts (GNU Octave^37^).

## Results and discussion

**Figure 1:**
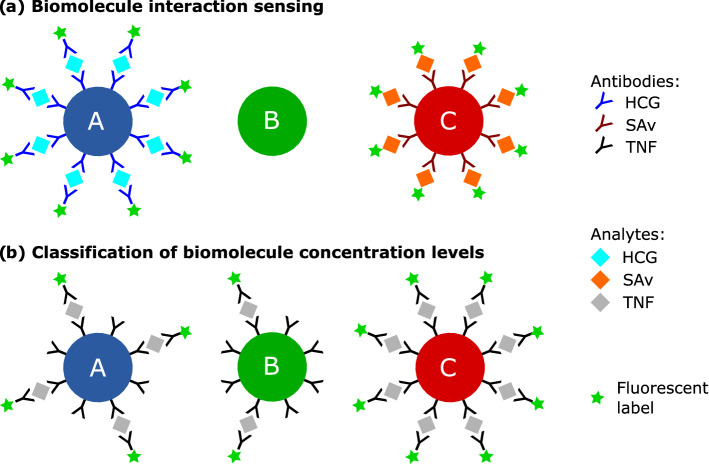
Study design based on surface-functionalized ‘red’ emitting LT-encoded beads, i.e., codes A, B, and C. (**a**) Biomolecule interaction sensing and (**b**) classification of different concentration levels of biomolecules using ‘green’ emitting reporter molecules.

We assessed the potential of our LT-encoding and multiplexing concept for LT-FCM in the time domain in two steps. First, discrimination of LT-encoded beads carrying different bead-bound biomolecules (HCG and SAv) was shown (see Fig. 1, panel a)) and secondly, the classification of the amount of bead-bound biomolecules was exemplarily demonstrated for TNF-α (see Fig. 1, panel b)). The discrimination of the carrier beads is based on fluorescence LT measurements in the ‘red’ channel in the wavelength region > 630 nm. The analyte concentration is assessed based on the fluorescence intensity detected in the ‘green’ channel within a spectral window determined by the fluorescence band of the label AlexaFluor488 centred at 520 nm. For these studies, PMMA beads of almost the same size have been chosen and three different LT codes A, B, and C were realized utilizing two organic dyes with different decay kinetics and their mixtures. Prior to the LT-FCM studies, the LT-encoded beads were characterized by fluorescence measurements of ensembles of beads in suspension. Subsequently, LT-FCM measurements with the different ‘red’ emitting bead codes surface-modified with target-specific ‘green’ emitting antibodies (see Fig. 1, Table 1, and ‘Sample Preparation’ section) were carried out.

**Table 1:** List of all samples used, and study design based on surface-functionalized LT-encoded beads, i.e., codes A, B, and C.

Sample	LT code	Analyte	Label	Description
**a) Biomolecule interaction sensing**				
A1	A	HCG	antiHCG 488	HCG pos
A2		–		HCG neg
B	B	–	–	Free
C1	C	SAv488	–	SAv pos
C2		–	–	SAv neg
M	A	HCG	antiHCG 488	Mixture (pos)
	C	SAv488	–	
**b) Classification of concentration levels of biomolecules**				
A0	A	–	Mab11 488	TNF-α neg
B0	B			
C0	C			
AM	A	TNF-α		TNF-α medium
BL	B			TNF-α low
CH	C			TNF-α high

(a) Biomolecule interaction sensing. (b) Classification of different concentration levels of biomolecules.

### Ensemble measurements of beads in suspension—fluorescence characteristics of bead-based LT codes

**Figure 2:**
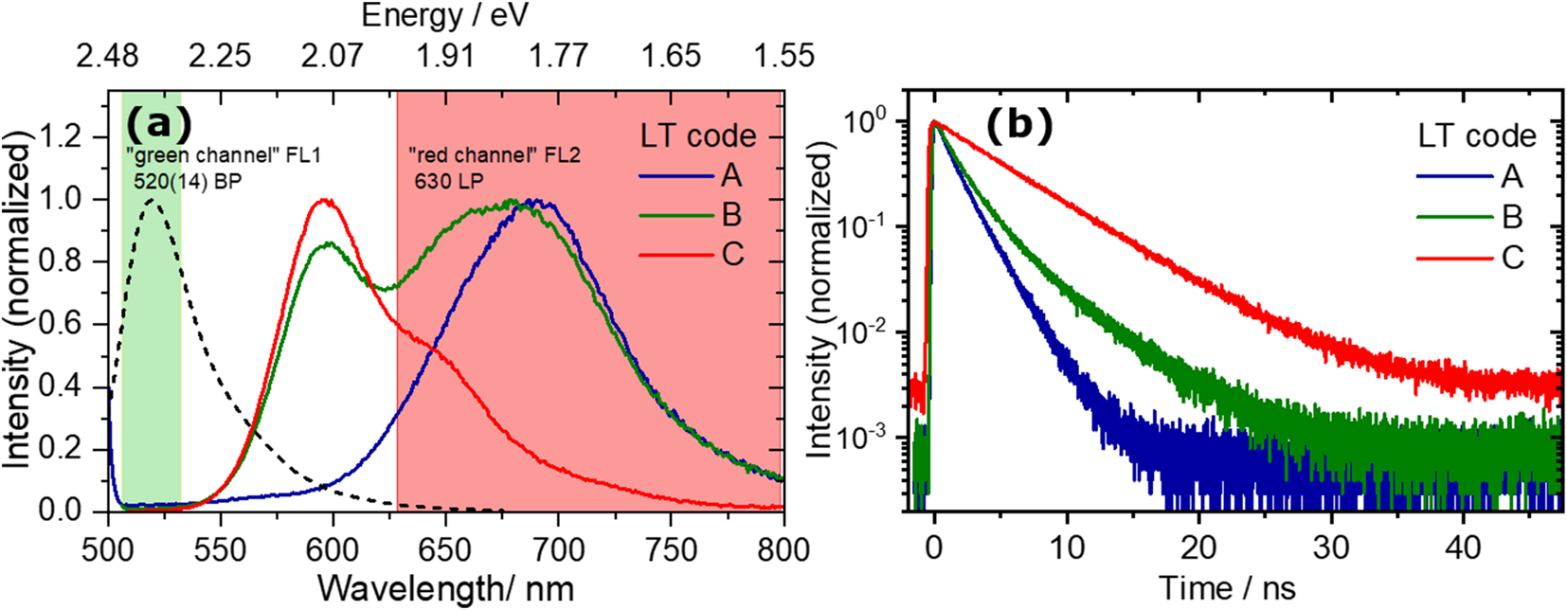
Fluorescence emission spectra (**a**) and fluorescence decay curves (**b**) of suspensions of the LT-encoded beads excited at 488 nm. The emission spectra of the beads cover the spectral range from around 550–800 nm. The area highlighted in red (‘red’ channel) indicates the spectral window used for LT detection. The green area (‘green’ channel) represents the spectral window used for the readout of a ligand signal, here the emission spectrum of the ligand label AlexaFluor 488^38^ (black dashed line) is shown for comparison. The spectral range for the detection of the fluorescence decay curves was set with long pass filters: 645 nm cut-off for codes A and B and 590 nm for code C (for intensity reasons only).

Fluorescence emission spectra of the bead ensembles in suspension of all three LT codes A, B, C, and the fluorescent label AlexaFluor488 are displayed in Fig. 2a. All samples were excited at 488 nm to match the excitation/emission conditions of the LT-FCM setup^31^. The fluorescence emission spectra of the LT codes cover a wavelength range from around 550 to 800 nm which allows for straightforward detection of all three codes with a long-pass filter. The spectral window covered by the long-pass used in the LT-FCM setup is indicated in Fig. 2a by the area highlighted in red. The area highlighted in green represents the wavelength region intended for the readout of a spectrally distinguishable ligand signal for analyte quantification utilizing a fluorescent reporter. This enables LT discrimination of differently encoded beads and quantification from measured fluorescence intensities of the reporter in different spectral windows.

As can be seen from the emission spectra in Fig. 2a, the emission of the ‘green’ label tails into the spectral region used for lifetime detection. However, according to control experiments performed earlier, the spectral overlap is too small and the fluorescence intensity contribution from AlexaFluor 488 is too small to hamper the reliable determination of the encoding parameter LT. Moreover, the readout of the LT codes at longer wavelengths than used for the detection of the fluorescence intensity from the ligand elegantly circumvents a possible impact of e.g. potentially disturbing energy transfer processes (e.g. fluorescence or Foerster resonance energy transfer,—acronym FRET) between ligands and dye molecules in the core of the LT-encoded beads. In addition, FRET between the dye molecules in the bead core and the ligand reporter molecules is unlikely due to the protein layer on the bead surface which is around 10 nm thick. This distance is too large for efficient FRET processes^1^.

The fluorescence decay curves of ensembles of the three LT bead codes in suspension are shown in Fig. 2b. Excitation was at 488 nm and the emission was detected with the monochromator set to zeroth diffraction order and a long-pass filter to simulate the measurement conditions in the LT-FCM setup^31^. The decay kinetics of the bead ensembles are clearly distinguishable in these measurements with a large number of counts per decay curve. These fluorescence decay curves as well as the derived LT values given in Table 2 highlight the considerable differences between the codes which is a prerequisite for effective LT-discrimination^31^.

**Table 2:** Overview of the measured LTs of the codes used.

Code	Bead diameter (µm)	Lifetimes/ns		
		LT-encoded bead ensembles	Study on biomolecule interaction sensing	Study on classification of concentration levels
		τ_int_ (reference)^31^	τ_FCM-s_*^)^	τ_FCM-c_^+^^)^
A	6.5	1.72	2.0 ± 0.7	3.0 ± 1.7
B	6.3	2.71	3.6 ± 0.7	4.4 ± 0.9
C	8.8	5.54	5.1 ± 0.8	6.0 ± 0.8

The fluorescence reference LTs are derived from measurements of bead suspensions in cuvettes. They were obtained with the lifetime spectrometer as intensity-averaged mean values τ_int_ from multi-exponential decay fits to the data. The lifetimes τ_FCM-s_ and τ_FCM-c_ derived from the study on biomolecule interaction sensing and from the study on classification of concentration levels, respectively, were extracted from LT-FCM measurements.

*^)^ measured τ_FCM-s_ in the study on biomolecule interaction sensing.

^+^^)^ measured τ_FCM-c_ in the study on classification of different concentration levels of biomolecules.

As follows from Table 2, the fluorescence lifetimes $\tau_{{\text{FCM-s}}}$ and $\tau_{{\text{FCM- c}}}$ vary between the two studies on biomolecule interaction sensing and on classification of biomolecule concentration levels (see Fig. 1a,b, respectively) as slightly different parameters like signal intensity/photon count number, applied pressure, integration time range, and bin width were used for the respective measurements. As previously demonstrated, different experimental conditions can influence the FCM lifetimes obtained for the two series^31^. However, as long as a discrimination of codes is possible, the exact determination of LT values is not relevant for LT multiplexing and barcoding applications.

### Single bead measurements—simultaneous detection of bead code and ligand emission

**Figure 3:**
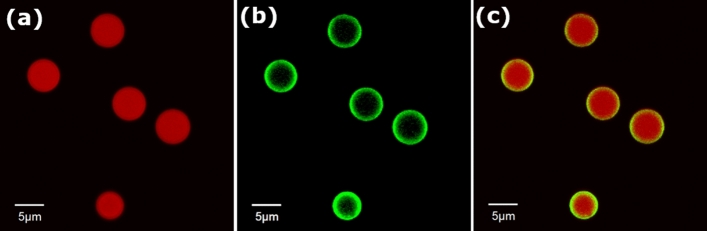
Exemplary, false-colour confocal laser scanning microscopy images of PMMA beads loaded with code B. (**a**) Spectral region of the bead core (code). (**b**) Spectral region of the fluorescently labelled ligand (TNF-α detection). (**c**) Overlay of both spectral channels. Excitation wavelengths: 488 and 543 nm, the different spectral channels were defined by optical filters (detection settings: channel 1 was defined by an emission dichroic mirror SDM560 combined with a variable band pass filter position 495 nm and a filter range of 100 nm, and channel 2 by a barrier filter BA650IF).

In our proof-of-principle studies on biomolecule interaction sensing with LT-FCM, fluorescent reporter molecules were attached to the surface of the respective LT-encoded beads by binding of the fluorophore-labelled targets to their surface ligands. Successful biomolecule capture and spectral discrimination between the bead code emission and the fluorescence signal from the reporter-labelled biomolecules attached to the bead surface was demonstrated by fluorescence microscopy. Exemplary CLSM images in Fig. 3 clearly illustrate the distinction between bead code B (core), and the fluorescence originating from surface-bound TNF-α labelled with the detection antibody Mab11-AlexaFluor488 as fluorescent reporter. The corresponding overlay of the fluorescence signals in both channels is shown in the right panel of Fig. 3. The spectrally different core codes and the ligand label are clearly visible. This proves that the emission spectra of the LT codes and the reporter fluorescence do not spectrally overlap. To demonstrate our concept on biomolecule interaction sensing and detection, one of the two emission channels of the LT-FCM is used for the detection of the LT-codes (red highlighted area in Fig. 2a). The second channel (green highlighted area in Fig. 2a) can read-out the fluorescence intensity of the reporter-conjugated biomolecule-specific ligands, thereby providing a measure for the amount of analyte in the sample. In this detection scheme, the achievable degree of multiplexing is determined by the number of discriminable LT codes.

### Time domain flow cytometry with nanosecond resolution

The LT-encoded beads were subsequently used in a proof-of-principle study on simultaneous spectral and LT discrimination with a previously described novel (prototype) LT-FCM setup^31^. Thereby, we focussed on two simple scenarios based on three LT codes. First, we consider sensing and discrimination of bead-bound biomolecules, and secondly, the classification of different concentration levels of bioanalytes, see Fig. 1.

### Biomolecule interaction sensing

For the study on biomolecule interaction sensing, capture antibodies for HCG were attached to code A beads and SAv capture antibodies to code C beads (see section *Sample Preparation* for more details). Positive control samples A1 and C1 were prepared by adding HCG antigen or SAv, respectively. HCG was detected fluorometrically using AlexaFluor488-labelled HCG detector antibodies added to the bead suspensions. In the case of SAv labelled with AlexaFluor488, direct detection of the bead-bound target was feasible. The negative control samples A2 and C2 did not contain HCG and SAv. Additionally, a mixed sample M was prepared containing all three LT codes and both dye-labelled targets, by mixing beads with the capture antibodies attached in a new vial. Subsequently, HCG and SAv as well as fluorophore-labelled HCG detector antibodies were added to this mixed sample. Additionally, code B-beads, which are not conjugated to a biomolecule at this stage, were included to demonstrate that also an additional LT code can be used. An overview of all samples is given in Table 1.

**Figure 4:**
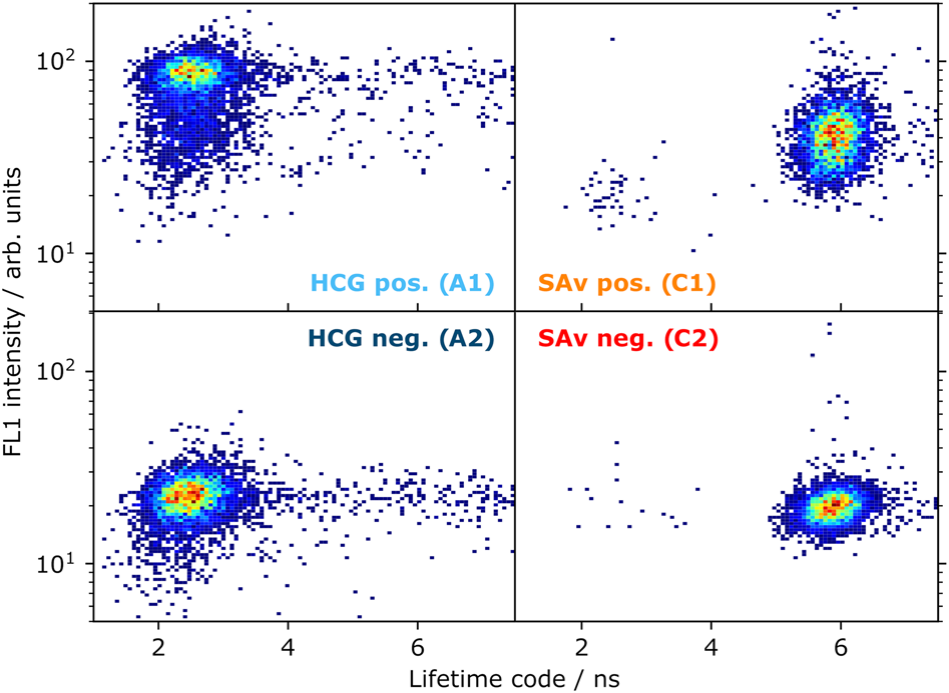
Control samples A1/2 and C1/2 used for the experiment on biomolecule interaction sensing with HCG and SAv. Pseudocolour dotplots of ligand fluorescence intensity and measured lifetime of the individually prepared and measured positive (A1 and C1) and negative control samples (A2 and C2) for both analytes (blue bins: low number of events; red bins: high number of events).

At first, LT-FCM measurements were performed with the single-analyte samples A1, A2 and C1, C2. Figure 4 shows pseudocolour dotplots from four separate measurements of the ligand fluorescence intensity in the ‘green’ channel and the lifetime measured for each bead. The LT codes A and C can clearly be distinguished in the LT-FCM measurements, notwithstanding the fact, that for the code A with the shorter lifetime, some events exhibit significantly longer lifetimes in our experiments. This fraction is relatively low, and the deviation is caused by an increased background signal level that artificially shifts the obtained lifetimes to higher values. The fluorescence intensity from the ligands differs between the positive and negative control samples by either a factor of around 5 in the case of HCG, or a factor of 2 for SAv, respectively.

**Figure 5:**
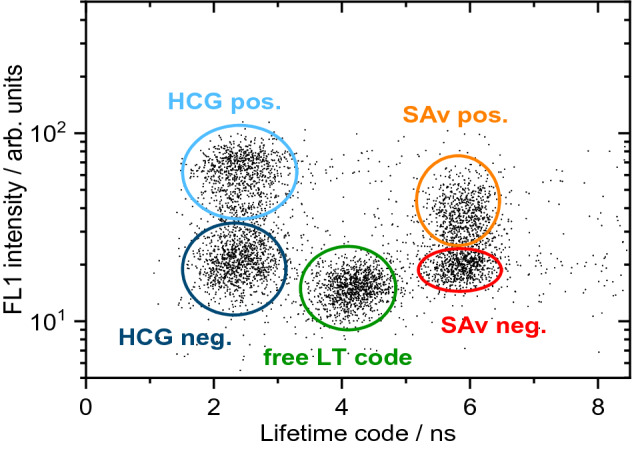
Dotplot of ligand fluorescence intensity and measured lifetimes obtained for a mixture of the samples A1 and A2, B as well as C1 and C2.

Results from a further LT-FCM experiment performed with a mixture of the individual samples A1, A2, B, C1, and C2 are given in Fig. 5. The resulting dotplot clearly reveals the feasibility of the LT encoding approach for bead-based FCM analysis. The beads with code B can be used for the detection of a third analyte, e.g. in a triplex assay. The very slight difference in the signal intensities of the fluorescent label of HCG observed for the independently performed experiments shown in Figs. 4 and 5 may provide a hint for sample aging, i.e., a slight degradation of the samples with time.

**Figure 6:**
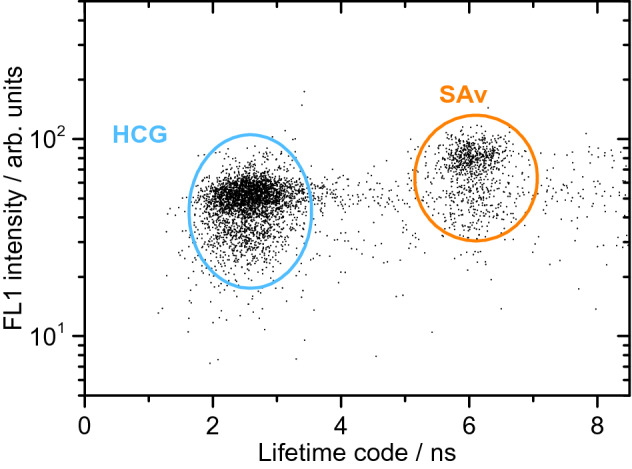
Dotplot of ligand fluorescence intensity and measured lifetimes of the assay-like sample M demonstrating the possibility for LT-based discrimination between LT codes with attached fluorescent bioligands.

A final LT-FCM scenario resembles the prospective application in bead-based assays, where beads with fluorescence LT codes and the respective target-specific fluorophore-labelled capture antibodies are simultaneously present in a sample. Figure 6 shows the dotplot of the ligand fluorescence intensity in the ‘green’ ligand channel FL1 and the measured lifetime for the assay-like sample M. This experiment confirms that LT-encoded beads can be utilized in multi-component assays replacing or complementing the known approaches with spectral encoding.

### Classification of concentration levels of bioanalytes

In further studies, we assessed the possibility for analyte quantification in our LT-based multiplexing approach. For this purpose, we semi-quantitatively determined different concentration levels of the representatively chosen analyte TNF-α in a single-target assay containing different concentrations of this analyte (see lower part of Table 1).

**Figure 7:**
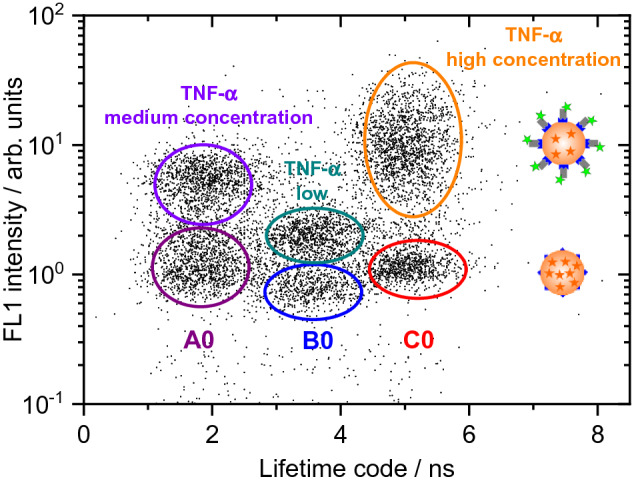
Dotplot of a model for a single-target immunoassay performed on a mixture of three different LT-encoded bead populations. The signal detected in a spectrally distinguishable intensity channel (‘green‘ channel: 520(14) nm bandpass), originating from the ligand fluorescence, is a measure of the analyte concentration.

The dotplot obtained for a mixture of three control samples (A0, B0, and C0) and the corresponding samples containing TNF-α at high (CH), medium (AM), and low (BL) concentration levels is displayed in Fig. 7. The signal in the ‘green’ ligand channel originates from the TNF-α detection antibody Mab11-AlexaFluor488 and provides a measure for the concentration of this biomarker. The concentration levels of negative or positive signals are obtained by certain concentration cut-offs.

Figure 7 clearly confirms that all six bead populations can be separated. This experiment can pave the way for biomolecule quantification utilizing the technical possibility of simultaneous spectral and LT discrimination with the novel LT-FCM setup operating in the time domain.

## Conclusion and Outlook

We assessed the suitability of lifetime (LT)-encoded beads to act as a platform for biomolecule sensing and concentration level classification using a recently developed novel luminescence lifetime flow cytometer (LT-FCM) setup operating in the time domain. Using this instrument and a set of LT-encoded beads bearing target-specific surface ligands, we could demonstrate that by including a photon counting detector for time-resolved measurements, an additional dimension in parameter space can be added to flow cytometric measurements. Based on the presented proof-of-concept multiplexing studies with LT-encoded PMMA carrier beads surface-functionalized with different bioligands, we confirmed the feasibility of the simultaneous readout of LT codes and ligand fluorescence intensities. Our study underlines the applicability of LT-FCM with nanosecond time resolution for future developments in bead-based sensing of biomolecular interactions in suspension assays. In the future, the number of LT codes that determines the achievable degree of multiplexing can be enhanced by expanding the time scale. Moreover, depending on the desired application, the LT-FCM design can be further developed, incorporating e.g., more light sources and detectors, to enable advanced multiplexing schemes.
